# Understanding loneliness and depression in psychiatry professionals: insights from a national survey of trainees and practitioners

**DOI:** 10.1186/s40359-025-03050-y

**Published:** 2025-07-01

**Authors:** Jarurin Pitanupong, Warut Aunjitsakul, Kanthee Anantapong

**Affiliations:** Department of Psychiatry, Faculty of Medicine, Prince of Songkla University, Hat Yai, 90110 Songkhla Thailand

**Keywords:** Loneliness, Psychiatrists, Internship and residency, burnout, professional, Stress, psychological

## Abstract

**Background:**

This research investigated the prevalence of loneliness and its associated factors. These included depression and attitudes towards social support and work among psychiatry trainees as well as practitioners.

**Methods:**

From January to February 2023, this cross-sectional study used an online survey to gather data from Thai psychiatry trainees and psychiatrists. The survey included: demographic and work-related questions, assessments of social support and work perceptions; the Revised UCLA Loneliness Scale (Thai version), and the Patient Health Questionnaire-9 (PHQ-9, Thai version). Descriptive statistics were used for initial analysis, while multiple logistic regression was employed to identify loneliness-associated factors.

**Results:**

Out of 225 participants, 52 were psychiatry trainees (23.1%), and 173 were psychiatrists (76.9%); with a median age of 34 years (interquartile range: 30–42). The survey revealed that 15.6% of participants experienced high levels of loneliness, with 9.6% of trainees and 17.3% of psychiatrists reporting this condition. No statistically significant differences in loneliness levels between trainees and practitioners were found. Multivariate analysis indicated that higher loneliness was linked to less control over work schedules and lower levels of family support.

**Conclusions:**

Addressing factors, such as control over work schedules and family support, could be crucial in mitigating loneliness among mental health professionals.

## Background

Mental health problems among mental health professionals, including psychiatrists, are often under-recognized and can have a significant impact on both their personal lives and professional competency [1, 2]. Research assessing mental health conditions along with related factors among physicians and psychiatrists is growing [2, 3, 4, 5]; wherein, loneliness is being mentioned as one factor associated with mental health. This is a subjective distress experienced when individuals feel alone, lack meaningful relationships and lack companionship, regardless of their social network presence [6]. One prior study reported the prevalence of loneliness among practicing physicians as 43%, and it was associated positively with burnout or emotional exhaustion, fatigue, thoughts of suicide and depression [7, 8]. Additionally, more intense loneliness was identified in female physicians in addition to those under 40 years of age [9].

In Thailand, there are only 0.72 psychiatrists per 100,000 people [10], a number deemed critically inadequate for meeting mental health needs. As a result, Thai psychiatrists have reported a diminished quality of life (QoL) [11]. Additionally, from 2011 until 2018, burnout rates among Thai psychiatrists surged from 17.1 to 49.3% [12]. Recent research highlights that 12.4% of Thai psychiatrists and psychiatry trainees are experiencing depression, with rates being 13.9% among psychiatrists and 7.7% among trainees. These levels of depressive symptoms align closely with findings from studies conducted on psychiatrists in North America [13].

Factors significantly linked to depression among psychiatrists include: being female, being in residency or early in one’s career, working in a non-academic setting, handling a higher patient load, working more than 50 h per week, dissatisfaction with work and the work environment and a lack of social support [13]. Furthermore, recent research has highlighted loneliness as a key factor contributing to depression among Thai psychiatrists [14]. Despite this, the relationship between loneliness and emotional distress, burnout and depression remains underexplored. Therefore, this study aimed to address this gap by assessing the prevalence of loneliness among psychiatry trainees and psychiatrists in Thailand; additionally, it also examined how work-related factors are associated with loneliness. The results are intended to guide stakeholders in developing strategies to address loneliness and enhance the mental well-being of Thai psychiatrists and trainees.

## Methods

Adhering to the ethical guidelines outlined in the Declaration of Helsinki, this research received approval from the Human Research Ethics Committee; Faculty of Medicine: Prince of Songkla University (REC.65-488-3-1). The study was conducted as a cross-sectional, online survey; between January and February 2023.

### Participants

Approximately 123 psychiatry trainees and 499 psychiatrists, all members of the Royal College of Psychiatrists of Thailand, were grouped via the official social media channels of the Thai Royal College of Psychiatrists. To facilitate data collection and management, it aimed to reach all available Thai psychiatry trainees and psychiatrists by announcing the study through official social media platforms, such as Facebook and the Line application (a freeware instant messaging app developed by LY Corporation). These applications are widely used daily by the Thai population, including psychiatric residents and psychiatrists. Eligible participants were those aged between 20 and 70 years, who were proficient in the Thai language.

To determine the appropriate sample size, we initially used the reported prevalence of loneliness among psychiatrists from a previous study: this being 15.6% [14]. We then utilized the ‘n.for.survey’ function from the Epicalc package in the R program to calculate the sample size for a finite population. With a margin of error (delta) at 0.04, an alpha level of 0.05, and a population size of 622, the calculation indicated that a minimum sample size of 213 participants was required.

### Data collection

Participants were recruited through the official social media channels of the Thai Royal College of Psychiatrists. The survey was promoted on these platforms using a poster containing a QR code and/or a link. The poster included a brief overview of the study’s purpose and background. To boost response rates, we reposted the survey biweekly on social platforms over a period of two months. By agreeing to participate, individuals could access the questionnaire via the provided link or QR code. In line with strict confidentiality policies, the Ethics Committee waived the requirement for written consent, informing participants that they could withdraw from the survey at any time without providing a reason.

### Measurements

Demographic information: such as gender, age, marital status, number of children as well as history of psychiatric and physical illnesses, was gathered from the participants. Additionally, work-related data were collected, including type of psychiatrist (child and adolescent psychiatrist versus general psychiatrist), career status (psychiatry trainees versus psychiatrists), type of workplace (psychiatric hospital, general/community/private hospital, clinic or academic medical school) and type of patient care (outpatient versus both outpatient and inpatient care). Other variables included: years of experience as a psychiatry trainee or psychiatrist, the number of patients seen per day, clinical hours per week, night shifts per month, paid days off per month and instances of patient suicide. Furthermore, we assessed the perceived social support as poor or strong from family, peers, and the head of the department. To evaluate perceptions of work, participants used a visual analog scale (VAS), ranging from 1 to 10 (extremely low to extremely high), to rate their work-related stress, perceived ability to control their work schedule and satisfaction with income and work.

### Loneliness

The Thai version of the 6-item Revised UCLA Loneliness Scale (RULS-6) is a self-administered questionnaire designed to assess loneliness. The 6-item version was chosen over the original 20-item version for its brevity and strong psychometric properties [15], making it especially suitable for online surveys with time-constrained healthcare professionals.

Participants rate each of the six items on a 4-point scale: 1 (never), 2 (rarely), 3 (sometimes) and 4 (always), with total scores ranging from 6 to 24. Higher scores indicate a greater level of loneliness. Scores were then categorized into three groups: ‘low’ for those below the 25th percentile, ‘average’ for scores between the 25th and 75th percentiles, and ‘high’ for those above the 75th percentile. The Thai version of the RULS-6 maintained its psychometric properties after translation having a Cronbach’s alpha of 0.83 reported in previous studies [15]. In this study, Cronbach’s alpha coefficient was 0.87, indicating good reliability.

### Depression

The Thai version of the Patient Health Questionnaire-9 (PHQ-9) is a self-administered tool used to assess depression, which consists of 9 items. Each item is rated on a 4-point scale: 0 (never), 1 (rarely), 2 (sometimes) and 3 (always), with total scores ranging from 0 to 27. The score ranges correspond to different levels of depression: 0–4 indicates no or minimal depression, 5–9 indicates mild depression, 10–14 indicates moderate depression, 15–19 indicates moderately severe depression, and 20–27 indicates severe depression. The PHQ-9 Thai version has been validated and shows acceptable psychometric properties for depression screening. It has a recommended cut-off score of nine or higher, offering a sensitivity of 0.53 and a specificity of 0.98, along with a Cronbach’s alpha of 0.79 [16]. In this current study, the PHQ-9 demonstrated a Cronbach’s alpha coefficient of 0.81: indicating good reliability.

### Statistical analyses

All data: including demographic information, work-related variables and other variables of interest, were analyzed using descriptive statistics. These included: proportions, means, standard deviations (SD), medians and interquartile ranges (IQR). Depending on the distribution of the variables, the Mann-Whitney U test, Fisher’s exact test, or Chi-square test were employed. To examine the association between loneliness scores and various demographics, work-related factors as well as other variables of interest, multiple logistic regression analyses were conducted using the R software program version 4.3.1 [17]. Confidence intervals (CIs) were calculated at a 95% level on a two-sided basis. We assessed multicollinearity using variance inflation factor (VIF), and found no significant issues (VIF < 5).

## Results

### Demographic characteristics

Table 1 provides additional demographic details of the study participants. A total of 225 individuals agreed to participate and completed the questionnaires: including 52 (23.1%) psychiatry trainees and 173 (76.9%) psychiatrists, resulting in a response rate of 36.2%. The median age (IQR) was 28 years (27, 29) for psychiatry trainees and 37 years (33, 44) for psychiatrists. Most participants were female (146, 64.9%), single or divorced (146, 64.9%), and had a history of physical (91, 40.4%) and psychiatric illnesses (19, 8.4%). The most reported physical conditions were allergies (10.2%) and dyslipidemia (7.6%), while the most frequent psychiatric conditions were depression (6.2%), anxiety disorder (1.4%), post-traumatic stress disorder (0.4%), and attention deficit hyperactivity disorder (0.4%). In terms of work-related characteristics most participants were general psychiatrists (178, 79.1%), employed at a medical school (105, 46.7%), worked 40 to 50 h per week (108, 48.0%) and had 6–8 days off per month (101, 44.9%).

**Table 1:** Demographic and work-related characteristics (*N* = 225)

Demographic characteristics	Total (%)	Psychiatrists	Psychiatry trainees
		(*N* = 173)	(*N* = 52)
**Gender**			
Male	79 (35.1)	64 (37.0)	15 (28.8)
Female	146 (64.9)	109 (63.0)	37 (71.2)
**Age (years)**			
Median (IQR)	34 (30, 42)	37 (33,44)	28 (27,29)
**Marital Status**			
Single/Divorce	146 (64.9)	99 (57.2)	47 (90.4)
Married	79 (35.1)	74 (42.8)	5 (9.6)
**Number of children**			
None	176 (78.2)	125 (72.3)	51 (98.1)
One	29 (12.9)	28 (16.2)	1 (1.9)
More than one	20 (8.9)	20 (11.6)	0 (0.0)
**Physical illness**			
No	134 (59.6)	100 (57.8)	34 (65.4)
Yes	91 (40.4)	73 (42.2)	18 (34.6)
**Psychiatric illness**			
No	206 (91.6)	159 (91.9)	47 (90.4)
Yes	19 (8.4)	14 (8.1)	5 (9.6)
**Experience as a psychiatrist (years)**			
Median (IQR)	6 (3, 14)	9 (5,15)	2 (1,3)
**Workplace**			
Medical school	105 (46.7)	60 (34.7)	45 (86.5)
Psychiatric hospital	24 (10.7)	17 (9.8)	7 (13.5)
Other hospital **(g**eneral/community/ private hospital/clinic)	96 (42.7)	96 (55.5)	0
**Position**			
General psychiatrist	178 (79.1)	138 (79.8)	40 (76.9)
Child and adolescent psychiatrist	46 (20.4)	35 (20.2)	11 (21.2)
No answer	1 (0.4)	0	1 (1.9)
**Work type**			
Only outpatient	22 (9.8)	22 (12.7)	0 (0.0)
Outpatient and inpatient	203 (90.2)	151 (87.3)	52 (100)
**Number of patients per day**			
Median (IQR)	20 (10, 30)	25 (14,35)	10 (6,15)
**Working hours per week**			
< 40	57 (25.3)	51 (29.5)	6 (11.5)
40–50	108 (48.0)	80 (46.5)	28 (53.8)
> 50	59 (26.2)	41 (23.7)	18 (34.6)
No answer	1 (0.4)	1 (0.6)	0
**Number of shifts per month**			
Median (IQR)	6 (4, 10)	6 (2.8,10)	5.5 (4,9.2)
**Number of days off per month**			
0–2	21 (9.3)	13 (7.5)	8 (15.4)
3–5	48 (21.3)	39 (22.5)	9 (17.3)
6–8	101 (44.9)	68 (39.3)	33 (63.5)
8–10	46 (20.4)	44 (25.4)	2 (3.8)
> 10	9 (4.0)	9 (5.2)	0 (0.0)
**Duration of death in patients who committed suicide**			
None	125 (55.6)	74 (42.8)	51 (98.1)
Less than 1 month	6 (2.7)	6 (3.5)	0 (0.0)
1 month to 1 year	25 (11.1)	24 (13.9)	1 (1.9)
More than 1 year	69 (30.7)	69 (39.9)	0 (0.0)

IQR = Interquartile range

Table 2 presents the levels of loneliness, depression, satisfaction and quality of social support among psychiatric trainees and psychiatrists. No significant differences were found between the two groups; except for income satisfaction, which was notably lower among psychiatric trainees compared to psychiatrists.

**Table 2:** Level of depression, loneliness, satisfaction and quality of social support (*N* = 225)

Variables	Total (%)	Psychiatrists	Psychiatry trainees	Chi2 *P*-value
		(*N* = 173)	(*N* = 52)	
**Loneliness**				0.259
Low	0			
Average	190 (84.4)	143 (82.7)	47 (90.4)	
High	35 (15.6)	30 (17.3)	5 (9.6)	
**Depression**				0.329
Minimal / no	143 (63.6)	110 (63.6)	33 (63.5)	
Mild	63 (28.0)	46 (26.6)	17 (32.7)	
Moderate / severe	19 (8.4)	17 (9.8)	2 (3.8)	
**Level of income satisfaction**				
Median (IQR)	6.0 (5, 8)	7 (5,8)	4 (2.8,5.2)	< 0.001^a^
**Level of work satisfaction**				
Median (IQR)	7.0 (6, 8)	7 (6,8)	7 (6,8)	0.886^a^
**Level of work stress**				
Median (IQR)	6.0 (5, 8)	6 (4,7)	7 (5,8)	0.045^a^
**Ability to control work schedule**				
Median (IQR)	7.0 (5, 8)	7 (5,8)	6 (5,7)	0.023^a^
**Supporting person**				
Yourself	161 (71.6)	120 (69.4)	41 (78.8)	0.249
Chief	23 (10.2)	17 (9.8)	6 (11.5)	0.923
Friend	167 (74.2)	122 (70.5)	45 (86.5)	0.033
Partner	132 (58.7)	107 (61.8)	25 (48.1)	0.108
Family	92 (40.9)	69 (39.9)	23 (44.2)	0.691
Social media	10 (4.4)	7 (4.0)	3 (5.8)	0.701^b^
Others	8 (3.6)	8 (4.6)	0 (0.0)	0.203^b^
**Level of social support**				
Family				0.526^b^
Good	199 (88.4)	155 (89.6)	44 (84.6)	
Poor	12 (5.3)	8 (4.6)	4 (7.7)	
No answer	14 (6.2)	10 (5.8)	4 (7.7)	
Head of Department				0.089
Good	149 (66.2)	108 (62.4)	41 (78.8)	
Poor	26 (11.6)	22 (12.7)	4 (7.7)	
No answer	50 (22.2)	43 (24.9)	7 (13.5)	
Psychiatric friend				0.166
Good	178 (79.1)	132 (76.3)	46 (88.5)	
Poor	17 (7.6)	15 (8.7)	2 (3.8)	
No answer	30 (13.3)	26 (15.0)	4 (7.7)	
Physicians in other departments				0.762
Good	133 (59.1)	103 (59.5)	30 (57.7)	
Poor	35 (15.6)	28 (16.2)	7 (13.5)	
No answer	57 (25.3)	42 (24.3)	15 (28.8)	
Hospital administrative staff				0.173
Good	106 (47.1)	82 (47.4)	24 (46.2)	
Poor	69 (30.7)	57 (32.9)	12 (23.1)	
No answer	50 (22.2)	34 (19.7)	16 (30.8)	
Other colleagues				0.312^b^
Good	185 (82.2)	143 (82.7)	42 (80.8)	
Poor	20 (8.9)	17 (9.8)	3 (5.8)	
No answer	20 (8.9)	13 (7.5)	7 (13.5)	

IQR: Interquartile range, SD: Standard deviation, ^a^Mann-Whitney U test, ^b^Fisher’s exact test

### Loneliness and participant characteristics

According to RULS-6, most participants (190, 84.4%) reported an average level of loneliness, while 35 (15.6%) indicated a high level of loneliness. Among these, 9.6% (5/52) of psychiatry trainees and 17.3% (30/173) of psychiatrists reported a high level of loneliness. As shown in Table 3, there were no significant differences in participant characteristics, nor the presence of depression between those with average and high levels of loneliness.

**Table 3:** Demographic, depression, and work-related characteristics categorized by level of loneliness (*N* = 225)

Demographic Characteristics	Total (%)	Loneliness		Chi2 *P*-value
		Average	High	
		(*N* = 190)	(*N* = 35)	
**Gender**				0.935
Male	79 (35.1)	66 (34.7)	13 (37.1)	
Female	146 (64.9)	124 (65.3)	22 (62.9)	
**Age (years)**				0.540^a^
Median (IQR)	34 (30, 42)	34 (30,42)	35 (30.5,42.5)	
**Marital Status**				0.491
Single/Divorce	146 (64.9)	121 (63.7)	25 (71.4)	
Married	79 (35.1)	69 (36.3)	10 (28.6)	
**Number of children**				0.164
None	176 (78.2)	145 (76.3)	31 (88.6)	
One or more	49 (21.8)	45 (23.7)	4 (11.4)	
**Physical illness**				0.806
No	134 (59.6)	112 (58.9)	22 (62.9)	
Yes	91 (40.4)	78 (41.1)	13 (37.1)	
**Psychiatric illness**				0.745^b^
No	206 (91.6)	173 (91.1)	33 (94.3)	
Yes	19 (8.4)	17 (8.9)	2 (5.7)	
**Experience as a psychiatrist (years)**				0.364^a^
Median (IQR)	6 (3, 14)	6 (3,13)	7 (4,15)	
**Workplace**				0.438
Medical school	105 (46.7)	92 (48.4)	13 (37.1)	
Psychiatric hospital	24 (10.7)	19 (10.0)	5 (14.3)	
Others	96 (42.7)	79 (41.6)	17 (48.6)	
**Position**				1
General psychiatrist	178 (79.5)	150 (79.4)	28 (80.0)	
Child and adolescent Psychiatrist	46 (20.5)	39 (20.6)	7 (20.0)	
**Work type**				1^b^
Only outpatient	22 (9.8)	19 (10.0)	3 (8.6)	
Outpatient and inpatient	203 (90.2)	171 (90.0)	32 (91.4)	
**Number of patients per day**				0.513^a^
Median (IQR)	20 (10, 30)	20 (10,30)	20 (10,40)	
**Working hours per week**				0.802
< 40	57 (25.4)	47 (24.9)	10 (28.6)	
*≥* 40	167 (74.6)	142 (75.1)	25 (71.4)	
**Number of shifts per month**				0.495^a^
Median (IQR)	6 (4, 10)	6 (4,10)	6 (4,9)	
**Number of days off per month**				1
8 or less	170 (75.6)	144 (75.8)	26 (74.3)	
> 8	55 (24.4)	46 (24.2)	9 (25.7)	
**Death of patients who committed suicide**				0.447
None	125 (55.6)	103 (54.2)	22 (62.9)	
Presence	100 (44.4)	87 (45.8)	13 (37.1)	
**Work position**				0.259
Psychiatrist	173 (76.9)	143 (75.3)	30 (85.7)	
Psychiatry trainees	52 (23.1)	47 (24.7)	5 (14.3)	
**PHQ-9 score**				0.162^b^
< 9	197 (87.6)	169 (88.9)	28 (80)	
*≥* 9	28 (12.4)	21 (11.1)	7 (20)	

IQR: Interquartile range, ^a^Mann-Whitney U test, ^b^Fisher’s exact test

### Loneliness and perceptions towards working conditions and social support

Table 4 indicates statistically significant differences between participants having average and high levels of loneliness regarding work satisfaction, work stress, ability to control work schedules and level of family support; with *p*-values of < 0.001, 0.003, < 0.001 and 0.009, respectively.

**Table 4:** Level of work and social support categorized by level of loneliness

Variables	Total (%)	Loneliness		*P*-value
		Average	High	
		(*N* = 190)	(*N* = 35)	
**Level of income satisfaction**				
Median (IQR)	6 (5, 8)	7 (5,8)	6 (3,7.5)	0.115^a^
**Level of work satisfaction**				
Median (IQR)	7 (6, 8)	7 (6,8)	6 (5,7)	< 0.001^a^
**Level of work stress**				
Median (IQR)	6 (5, 8)	6 (4,7)	7 (6,8)	0.003^a^
**Ability to control work schedule**				
Median (IQR)	7 (5, 8)	7 (5,8)	5 (3,6.5)	< 0.001^a^
**Supporting person**				1^b^
No support needed	9 (4.0)	8 (4.2)	1 (2.9)	
Required other person	216 (96.0)	182 (95.8)	34 (97.1)	
**Level of social support**				
Family				0.009^b^
Good	199 (88.4)	173 (91.1)	26 (74.3)	
Poor/no answer	26 (11.6)	17 (8.9)	9 (25.7)	
Other colleagues				0.633^b^
Good	216 (96.0)	183 (96.3)	33 (94.3)	
Poor/no answer	9 (4.0)	7 (3.7)	2 (5.7)	

IQR: Interquartile range, ^a^Mann-Whitney U test, ^b^Fisher’s exact test

We further selected variables with *p-*values from the univariate analysis (Tables 3 and 4) that were lower than 0.2 for multivariate analysis. The initial model included these variables: number of children, PHQ-9 score, income satisfaction, work satisfaction, work stress, perceived control over work schedule and social support. In Table 5, the final model from multivariate analysis using multiple logistic regression, identified that the ability to control work schedules and level of family support were factors associated with high levels of loneliness.

**Table 5:** Factors associated with high level of loneliness

Factors^a^	Crude OR (95%CI)	Adjusted OR (95%CI)	*P*-value LR-test
**Ability to control work schedules** ^b^	0.63 (0.52,0.77)	0.62 (0.5,0.76)	< 0.001
**Level of social support**			
Family			0.007
Poor/no answer	Reference	Reference	
Good	0.27 (0.11,0.68)	0.25 (0.09,0.65)	

Note:

^a^The remaining candidate variables were excluded through a backward stepwise procedure as they did not achieve a *p*-value less than 0.05 in the multivariable model

^b^Perceived ability to control work schedules using self-rating from 0–10 (extremely low-extremely high)

## Discussion

This study represents the first national survey from Thailand having focused on examining the prevalence and contributing factors of loneliness among Thai psychiatry trainees and psychiatrists. The findings revealed that 9.6% of psychiatry trainees and 17.3% of psychiatrists reported experiencing high levels of loneliness. The primary factors associated with loneliness were the lower perceived ability to control work schedules as well as the lower level of family support.

Currently, there is limited evidence regarding the prevalence of loneliness among psychiatry trainees or psychiatrists. The 15.6% prevalence of loneliness observed in this study is relatively lower when compared to a previous study among practicing physicians in the United States of America; wherein, 43% reported experiencing loneliness [7]. These discrepancies may be attributed to differences in study measurements, work conditions, and the social or cultural characteristics of the participants. In Thailand, Buddhist beliefs and collectivist cultural norms significantly shape mental health perceptions and help-seeking behaviors [18, 19], potentially leading to underestimations of prevalence in this study.

Although, there were no significant differences in loneliness levels between psychiatric trainees and psychiatrists, this study found that 9.6% of psychiatric trainees and 17.3% of psychiatrists reported high levels of loneliness. One possible explanation for these findings is that psychiatric trainees often benefit from support networks comprising of peers and departmental staff during their training period. Most participants in this study worked in medical schools and general hospitals. In Thailand, psychiatrists and trainees at medical schools typically have lighter clinical workloads and greater access to academic and social support [20]. In contrast, many psychiatrists in general and psychiatric hospitals work more independently and may be geographically distant from family and friends; increasing their risk of social isolation and reduced support.

This study also identified good family support as a factor associated with lower levels of loneliness. This suggests that interventions aimed at reducing loneliness among mental health professionals should particularly focus on those whom have already graduated. Given that the need for social connection is fundamental [21], mental health professionals experiencing loneliness may be reluctant to seek help or access mental health care due to stigma or confidentiality concerns. Addressing this issue is crucial, as there may be a greater stigma attached to mental illness within this profession compared to the public or other fields [22, 23, 24]. Therefore, efforts to prevent mental health problems among mental health professionals warrants significant attention.

Allowing young psychiatrists to work in regions close to their hometowns and families can be beneficial, as it provides them with accessible community and family support. However, if this is not feasible, establishing a social support system could be an effective alternative. Although, evidence is limited for healthcare professionals, Cognitive Behavioral Therapy (CBT) based interventions, group support, and digital platforms have effectively reduced loneliness and depression in both the general population and college students [25, 26]. This may also be applicable for psychiatrists and psychiatry trainees. In Thailand, for instance, the: “Network of Early Career Psychiatrists of Thailand”, and regional psychiatrist clubs offer a platform for senior psychiatrists to support the new generation. These networks can help provide professional and social support to young psychiatrists, mitigating feelings of isolation and enhancing their overall well-being [20].

This study identified the ability to control work schedules as a significant factor related to loneliness (adjusted OR = 0.62; 95% CI: 0.50–0.76; *p* < 0.001). In other words, the predicted probability of experiencing high loneliness decreases substantially as the ability to control work schedules increases, while holding social support constant at the reference level. When the ability to control work schedules is at its lowest score (0), the predicted probability of experiencing high loneliness is 91%. As the score increases to 5, this probability drops to approximately 47% and further decreases to just 7% at the maximum control level (score = 10). A potential explanation is that limited control over work schedules may lead to feelings of powerlessness, reduced autonomy and helplessness. Such work-related loneliness can both directly and indirectly affect emotional exhaustion, burden, depression as well as work-life balance [27]. Additionally, restricted control over work schedules might negatively impact on personal time available for social activities and connections; thereby, increasing loneliness. The clinical workplace should be a source of renewal and inspiration, not just a place to expend energy. A supportive work culture encourages work-life balance through regular breaks, paid leave, flexible schedules, and support for personal interests outside of work [28]. Furthermore, empowering psychiatrists to have greater control over their work schedules could help fulfill basic psychological needs and human motivation. Consequently, this could potentially reduce workplace loneliness in addition to the associated risk of mental health problems or depression [21].

Finally, this study found no significant association between depression and loneliness levels. This finding contrasts with existing literature, which generally links loneliness with the onset of common mental health issues and depression within the general population [29]. For instance: previous research has shown that work-related loneliness can contribute to emotional exhaustion or burnout and depression [14, 27]. A possible explanation for the discrepancy in this study’s results could be due to the small sample size of participants having experienced both high levels of loneliness and depression (*n* = 7). Although, we attempted post-hoc analysis with different PHQ-9 severity thresholds, the limited sample size still hindered detection of significant associations. The low sensitivity (0.53) of the PHQ-9 cut-off at nine or higher might also have underestimated depression prevalence. As this may have misclassified some depressed participants as non-depressed, diagnostic interviews may be needed for accuracy. However, the high specificity (0.98) minimizes false positives, so most identified cases were likely true depression [16].

### Strengths and limitations

To our knowledge, this study is the first to explore loneliness and its associations with work-related data, perceptions towards work, perceptions of social support and depression in Thailand. However, several limitations should be noted. Firstly, the online survey, which had a response rate of 36.2%, may have excluded those less active on social media or experiencing loneliness, distress or depression. In turn this may have introduced selection bias and likely underestimated loneliness and depression levels. Consequently, our sample may not fully represent all psychiatry trainees and psychiatrists nationwide, limiting generalizability. The survey’s anonymity also prevented comparison between responders and non-responders. Secondly, despite assurances of anonymity and no impact on professional status, psychiatrists and trainees may have underreported loneliness or depression due to stigma and cultural barriers, again likely leading to underestimation [19, 22]. Thirdly, fewer psychiatry trainees participated than expected, reducing statistical power for some analyses, including subgroup analysis. Nonetheless, the study achieved its objectives.

Fourthly, the cross-sectional design prevents causal conclusions between loneliness and associated factors in this study. Lastly, the study may have overlooked some potential confounding variables related to social support. We did not gather information on current stressors that might influence loneliness, which could have provided additional context for the findings. This study focused on individual factors, such as socio-economic status, lifestyle and social context; however, it did not address mental health issues influenced by social or work-related conditions. Future research should incorporate workplace variables; including settings (urban vs. rural hospitals), safety and insurance or welfare benefits. Longitudinal and mixed-method studies are needed to clarify causality and better understand how personal and professional factors interact to influence loneliness and depression in psychiatric practice. Longitudinal design could also help establish the temporal relationships between loneliness, autonomy and mental health outcomes. Further exploration of factors like loneliness, assertiveness, and support; especially as affected by social or work conditions, can provide targeted interventions to reduce loneliness and distress among mental health professionals.

## Conclusion

Our survey revealed that 9.6% of psychiatry trainees and 17.3% of psychiatrists experienced high levels of loneliness. Key factors significantly associated with loneliness were the ability to control work schedules and family support. To address loneliness among psychiatry trainees and psychiatrists, it is important to enhance social and family support in addition to encouraging greater flexibility in work schedules.

## Data Availability

No datasets were generated or analysed during the current study.

## Declarations

### Declarations

We confirm that all methods were carried out in accordance with relevant guidelines and regulations.

### Primary field

General topics in psychiatry and related fields.

## Abbreviations

MDD: Major depressive disorder
PHQ-9: Patient Health Questionnaire-9
QOL: Quality of life
RULS-6: The 6-item Revised UCLA Loneliness Scale

## References

[1] White A, Shiralkar P, Hassan T, Galbraith N, Callaghan R. Barriers to mental healthcare for psychiatrists. Psychiatr Bull. 2006;30(10):382–4. 10.1192/pb.30.10.382

[2] Kar N. Mental health of the psychiatrists: A need for reflection. Odisha J Psychiatry. 2022;18(2):67–70. 10.4103/ojp.Ojp_3_23

[3] Chatlaong T, Pitanupong J, Wiwattanaworaset P. Sleep quality and burnout syndrome among residents in training at the faculty of medicine, Prince of Songkla university. Siriraj Med J. 2020;72(4):307–14. 10.33192/Smj.2020.41

[4] Pitanupong J, Jatchavala C. A study on the comparison of burnout syndrome, among medical Doctors in the restive areas and Non-Restive areas of the South Thailand insurgency. J Health Sci Med Res. 2018;36(4):277–89. 10.31584/jhsmr.201825

[5] Thamrongvisava S, Pitanupong J. The prevalence and associated factors of burnout syndrome among residents in training at faculty of medicine, Songklanagarind hospital. J Psychiatric Association Thail. 2018;63(4):309–20.

[6] Havens B, Hall M, Sylvestre G, Jivan T. Social isolation and loneliness: differences between older rural and urban Manitobans. Can J Aging. 2004;23(2):129–40. 10.1353/cja.2004.0022 15334813 10.1353/cja.2004.0022

[7] Ofei-Dodoo S, Ebberwein C, Kellerman R. Assessing loneliness and other types of emotional distress among practicing physicians. Kans J Med. 2020;13:1–5. 32047581 PMC7006831

[8] Ofei-Dodoo S, Mullen R, Pasternak A, Hester CM, Callen E, Bujold EJ, et al. Loneliness, burnout, and other types of emotional distress among family medicine physicians: results from a National survey. J Am Board Fam Med. 2021;34(3):531–41. 10.3122/jabfm.2021.03.200566 34088813 10.3122/jabfm.2021.03.200566

[9] Keiner C, Nestsiarovich A, Celebi J, Zisook S. Loneliness among medical students, physician trainees and faculty physicians. Acad Psychiatry. 2024;48(4):339–45. 10.1007/s40596-023-01780-y 37038044 10.1007/s40596-023-01780-y PMC10088703

[10] WHO. Psychiatrists working in mental health sector Geneva, Switzerland WHO. 2025 [Available from: https://www.who.int/data/gho/data/indicators/indicator-details/GHO/psychiatrists-working-in-mental-health-sector-(per-100-000).

[11] Kosulwit L. Mental health and quality of life among Thai psychiatrists. J Med Association Thail = Chotmaihet Thangphaet. 2015;98(Suppl 2):S28–37. 26211101

[12] Nimmawitt N, Wannarit K, Pariwatcharakul P. Thai psychiatrists and burnout: A National survey. PLoS ONE. 2020;15(4):e0230204. 10.1371/journal.pone.0230204 32315309 10.1371/journal.pone.0230204 PMC7173626

[13] Summers RF, Gorrindo T, Hwang S, Aggarwal R, Guille C, Well-Being. Burnout, and depression among North American psychiatrists: the state of our profession. Am J Psychiatry. 2020;177(10):955–64. 10.1176/appi.ajp.2020.19090901 32660300 10.1176/appi.ajp.2020.19090901

[14] Pitanupong J, Anantapong K, Aunjitsakul W. Depression among psychiatrists and psychiatry trainees and its associated factors regarding work, social support, and loneliness. BMC Psychiatry. 2024;24(1):97. 10.1186/s12888-024-05569-7 38317097 10.1186/s12888-024-05569-7 PMC10840266

[15] Wongpakaran N, Wongpakaran T, Pinyopornpanish M, Simcharoen S, Suradom C, Varnado P, et al. Development and validation of a 6-item revised UCLA loneliness scale (RULS-6) using Rasch analysis. Br J Health Psychol. 2020;25(2):233–56. 10.1111/bjhp.12404 31999891 10.1111/bjhp.12404

[16] Lotrakul M, Sumrithe S, Saipanish R. Reliability and validity of the Thai version of the PHQ-9. BMC Psychiatry. 2008;8:46. 10.1186/1471-244X-8-46 18570645 10.1186/1471-244X-8-46 PMC2443128

[17] R Development Core Team. R: a Language and environment for statistical computing. Vienna: R Foundation for Statistical Computing; 2012.

[18] Burnard P, Naiyapatana W, Lloyd G. Views of mental illness and mental health care in thailand: a report of an ethnographic study. J Psychiatr Ment Health Nurs. 2006;13(6):742–9. 10.1111/j.1365-2850.2006.01028.x 17087678 10.1111/j.1365-2850.2006.01028.x

[19] Zhang Z, Sun K, Jatchavala C, Koh J, Chia Y, Bose J, et al. Overview of stigma against psychiatric illnesses and advancements of Anti-Stigma activities in six Asian societies. Int J Environ Res Public Health. 2019;17(1). 10.3390/ijerph17010280 10.3390/ijerph17010280 PMC6981757 31906068

[20] Wannarit K, Pukrittayakamee P, Udomratn P. Current status of psychiatric care in Thailand. Taiwan J Psychiatry. 2023;37(3):103–12. 10.4103/tpsy.Tpsy_22_23

[21] Deci EL, Ryan RM. The what and why of goal pursuits: human needs and the Self-Determination of behavior. Psychol Inq. 2000;11(4):227–68. 10.1207/S15327965PLI1104_01

[22] Loch AA, Hengartner MP, Guarniero FB, Lawson FL, Wang YP, Gattaz WF, et al. The more information, the more negative stigma towards schizophrenia: Brazilian general population and psychiatrists compared. Psychiatry Res. 2013;205(3):185–91. 10.1016/j.psychres.2012.11.023 23266022 10.1016/j.psychres.2012.11.023

[23] Rossler W. The stigma of mental disorders: A millennia-long history of social exclusion and prejudices. EMBO Rep. 2016;17(9):1250–3. 10.15252/embr.201643041 27470237 10.15252/embr.201643041 PMC5007563

[24] Sharma P, Kar N. Attitudinal impediments in the practice of consultation-liaison psychiatry. Archives Indian Psychiatry. 2012;14(1):45–50. doi.

[25] Anchuri K, Steiner L, Rabet R, Craig-Neil A, San Antonio E, Ogundele OJ, et al. Interventions in ambulatory healthcare settings to reduce social isolation among adults aged 18–64: a systematic review. BJGP Open. 2024;8(4). 10.3399/BJGPO.2023.0119 10.3399/BJGPO.2023.0119 PMC11687274 38760060

[26] Kang B, Hong M. Digital interventions for reducing loneliness and depression in Korean college students: mixed methods evaluation. JMIR Form Res. 2024;8:e58791. 10.2196/58791 39264705 10.2196/58791 PMC11427852

[27] Becker WJ, Belkin LY, Tuskey SE, Conroy SA. Surviving remotely: how job control and loneliness during a forced shift to remote work impacted employee work behaviors and well-being. Hum Resour Manag. 2022;61(4):449–64. 10.1002/hrm.22102

[28] Razai MS, Kooner P, Majeed A. Strategies and interventions to improve healthcare professionals’ Well-Being and reduce burnout. J Prim Care Community Health. 2023;14:21501319231178641. 10.1177/21501319231178641 37246649 10.1177/21501319231178641 PMC10233581

[29] Mann F, Wang J, Pearce E, Ma R, Schlief M, Lloyd-Evans B, et al. Loneliness and the onset of new mental health problems in the general population. Soc Psychiatry Psychiatr Epidemiol. 2022;57(11):2161–78. 10.1007/s00127-022-02261-7 35583561 10.1007/s00127-022-02261-7 PMC9636084

